# Perceived Quality of Service in Primary Health Care Based on Google Maps Reviews Before, During, and After the COVID-19 Pandemic: Sentiment Analysis

**DOI:** 10.2196/70410

**Published:** 2025-09-23

**Authors:** Lili Aunimo, Andreea M Oprescu, Dmitry Kudryavtsev, Luis Munoz Saavedra, Maria del Carmen Romero Ternero

**Affiliations:** 1 Research Area of Digital Transition and Artificial Intelligence Haaga-Helia University of Applied Sciences Helsinki Finland; 2 Departamento de Arquitectura y Tecnología de Computadores Instituto de Ingeniería Informática Universidad de Sevilla Seville Spain

**Keywords:** primary health care, perceived quality of service, social media, user-generated content, sentiment analysis, COVID-19, Google Maps reviews, deep learning models

## Abstract

**Background:**

The COVID-19 pandemic caused many changes in primary health care systems in Europe. The fast adoption of telemedicine, the shift of health care resources to COVID-19–related tasks, and the tendency of patients to cancel their nonurgent appointments are some examples of these changes. Patient satisfaction is an important outcome of health care services, and the changes caused by COVID-19 in the system may have affected it. Google Maps reviews provide an important channel for patients to communicate about their experiences regarding the primary health care system.

**Objective:**

Drawing from research on social media data analytics and text mining, this study set out to investigate the changes in public sentiment regarding primary health care in Finland and Andalusia (Spain) before, during, and after the COVID-19 pandemic.

**Methods:**

We collected 55,043 Google Maps reviews from primary health care locations in Finland and Andalusia from January 1, 2013, to May 15, 2024. There are 604 primary health care locations in Finland and 1016 in Andalusia. The total number of Google Maps reviews collected was 12,247 for Finland and 42,796 for Andalusia. First, lexicon-based sentiment analysis using the open-source software AFINN was conducted for the Finnish- and Spanish-language datasets. Thereafter, transformer-based deep learning models for sentiment analysis were applied for both languages. The numeric user ratings and the results of the sentiment analysis were then analyzed. In addition, we conducted a word frequency analysis of the reviews.

**Results:**

There were important changes in the ratings and sentiment in the data for Andalusia. The ratings shifted from median 4 (IQR 3) before the COVID-19 pandemic to median 1 (IQR 2) during and median 1 (IQR 3) after the COVID-19 pandemic, on a scale from 1 to 5. The median of the sentiment values of the review texts shifted from neutral before the COVID-19 pandemic to –2 (IQR 0.055) or –1 (IQR 1) during and after the COVID-19 pandemic, depending on which sentiment analysis method was used. Interestingly, changes in numeric ratings and sentiment of the review texts in Finland were only minor, and the median values were the same during all 3 periods. Lexical analysis revealed changes in word frequencies across the periods, reflecting shifts in primary health care experiences during the pandemic, especially among the Spanish-language reviews.

**Conclusions:**

The change toward a more negative public discussion on primary health care in Andalusia during the COVID-19 pandemic was considerable. This can be observed both in the numeric user ratings and in the sentiment analysis of the review texts. However, the data for Finland show that the public discourse stayed mostly neutral or slightly positive. The findings have implications on the quality management procedures in primary health care and on the use of user-generated content as an additional information source.

## Introduction

### Background

COVID-19 impacted the perceived quality of all facets of health care services across many European countries [1]. The pandemic led to the postponement of nonurgent appointments and treatments [2], prioritization of COVID-19 cases, and patient-initiated cancellations due to fear of infection [3]. Consequently, primary health care use and outpatient visits declined significantly, with a reported 60% decrease in hospital outpatient visits in the United States by early April 2020 [4].

Although the pandemic was officially declared over on May 5, 2023 [5], its effects continue to impact health care services. Physical distancing measures influenced service delivery, distinguishing between procedures requiring physical intervention (eg, surgery) and consultative services feasible through telemedicine (eg, psychotherapy) [5]. The backlog of postponed treatments and the rapid adoption of telemedicine have hindered full system recovery [1,5].

All aspects of health care services have been influenced by the pandemic. However, in this research, we specifically focused on primary health care services. Primary health care centers serve as the first point of contact for patients and play a main role in community health. Given their important role, it is essential to examine how the pandemic has impacted the perceived quality of care and whether these changes have persisted over time.

While it is important to study the internal functioning of the primary health care system, it is also important to study the quality of the primary health care system from the point of view of the patients. It is known that patients’ satisfaction regarding primary health care service quality is an important indicator of the success of the treatment [1] and of the overall quality of health care [2]. Patient satisfaction is typically monitored via self-report questionnaires [3]. Sometimes, qualitative methods such as interviews are also used. These are pertinent methods, but to complement them, we propose a methodology in which freely available user-generated content (UGC) can be exploited to gain additional insights into patient satisfaction. User-generated data from Google Maps in the form of user reviews can be mined to assess user satisfaction regarding health care services over time.

Research using Google Maps review data for analyzing user sentiment regarding primary health care is scant. The work by Lee et al [4] presents an analysis of Google Maps reviews of one hospital in Taiwan. As stated in the work by Ruelens [5], the amount of UGC on this topic is increasing, in part due to the COVID-19 pandemic, but is yet to be explored to its full potential. This study aimed to use this resource and further investigate its potential. While there is research on exploiting UGC from other platforms such as Facebook, X (formerly known as Twitter), and discussion forums, research using Google Maps reviews to monitor user sentiment in health care is scant. However, exploiting all types of UGC in addition to user surveys is valuable because user-generated reviews may highlight the views of those users who typically do not answer surveys. Other benefits of exploiting UGC is that, once the sentiment monitoring system is built and running, it can be operated cost-effectively to monitor user sentiment in real time. Sentiment analysis can also address issues that are not asked in a survey but that are expressed in the free-form writing of the reviews, thus providing a potentially richer insight into customer sentiment. Moreover, analysis of textual data collected through surveys often involves expensive labor-intensive manual coding, and the results are typically reported at longer intervals. In addition, UGC may reflect the views of users while they are being served and not after the service, which is the case with surveys [4]. To the best of our knowledge, this is the first study to use sentiment analysis to compare opinions obtained from Google Maps reviews before and after the COVID-19 pandemic. Our study is also the first attempt to assess user sentiment in Finland and Spain before, during, and after the COVID-19 pandemic; therefore, this is also the first study that compared the opinions in 2 different countries with different regulations on the COVID-19 pandemic. To bridge the gap in patient sentiment analysis research addressing perceived service quality in primary health care before, during and after COVID-19 using user generated data, we addressed the following research questions:

1. What is the sentiment of patients regarding primary health care organizations?
2. Are there variations in the sentiment over time when compared with the time phases before, during, and after the COVID-19 pandemic?
3. How can sentiment analysis be used to monitor the sentiment of users?

This research aimed to investigate the changes in public sentiment regarding primary health care in Finland and Andalusia (Spain) before, during, and after the COVID-19 pandemic using UGC from Google Maps reviews. The second goal of this study was a methodological one as we also attempted to assess how to build such a data processing pipeline. The results are expected to contribute to the body of knowledge on both the impacts of the COVID-19 pandemic on health care services and the methods for collecting and analyzing data regarding perceived service quality in primary health care.

### Related Work

#### Patient Sentiment Analysis in Health Care

Sentiment analysis has been widely used to study UGC in various domains, including health care [6]. In the study by Ruelens [5], researchers evaluated the public satisfaction with health care systems by applying natural language processing and sentiment analysis techniques to a specific type of UGC: newspaper readers’ comments. As part of the sentiment analysis, they also evaluated differences between male and female commenters, as well as differences at the country level. The authors found that user comments reflected concerns about the accessibility, affordability, and quality of health care systems. In addition, they identified that personal experiences with the health care system played a significant role in its evaluation.

Another longitudinal study aimed to characterize patient sentiments across the United States over a 4-year period [7]. Regarding COVID-19 specifically, Tsai and Wang [8] studied people’s attitudes toward public health policies and events through Twitter opinion mining. To gain a deeper understanding of public sentiment, the collected tweets were meticulously annotated across a spectrum of sentiments, ranging from very negative and negative to neutral to positive and very positive. The researchers found that stay-at-home and social distancing policies caused a strong negative impact among the public. Ainley et al [9] used Twitter data to gain insights into people’s perceptions of health care during the pandemic in the United Kingdom. Thematic and sentiment analysis was used to parse Twitter data. Public opinion on telemedicine during the pandemic was studied in the work by Pool et al [10] and Pollack et al [11]. This type of care was found to be positively received by most of the public. Facebook reviews as UGC and machine learning techniques were used to provide insights into hospital service quality in Malaysia [12]. The authors built a sentiment analyzer and service quality classifier for the dimensions of tangibles, reliability, responsiveness, assurance, and empathy. They found that most of the reviews were positive. Empathy and interpersonal aspects of care were linked to positive sentiment, whereas tangible aspects such as waiting times and service efficiency were linked to negative sentiment.

#### Sentiment Analysis on Google Maps Reviews

Multiple studies have researched sentiment analysis on reviews published on Google Maps. These studies explore various applications, such as assessing customer satisfaction with restaurants [13,14], evaluating tourism and visitors [15,16], and analyzing public opinion on services and amenities. In the health care domain, the application of sentiment analysis to Google Maps reviews is an emerging field. In the study by Herng Leong and Dahnil [17], the authors conducted sentiment analysis to detail the information on user satisfaction regarding health care services. They implemented the Valence Aware Dictionary for Sentiment Reasoning model on Google review data and proposed their work as a solution to help health care managers enhance their service quality. An education and research hospital used a chatbot to analyze and respond to Google Maps reviews [18]. Sentiment analysis was conducted using several algorithms. They found that Google Maps reviews are an important source of feedback. However, as is common with real-world data, unbalances can occur within the dataset.

In addition to textual reviews, Google Maps also includes a rating system ranging from 1 to 5 points. In the study by Shin et al [13], in which Google Maps reviews and ratings of restaurants were analyzed, the authors identified as a limitation the inconsistency between ratings and the sentiment expressed in the reviews. Specifically, they observed that 4-point ratings were accompanied by negative reviews and lower ratings (≤3 points) were accompanied by positive reviews. As a solution, they classified reviews with a rating of ≥4 as positive and those with a rating of ≤3 as negative.

#### Perceived Service Quality in Health Care

Health care service quality as perceived by patients is a well-researched and important topic. Perceived service quality may also be referred to as *patient satisfaction* or even *patient experience*. The overall role of patient satisfaction in determining service quality in health care has been growing during the last decades, and more traditional criteria such as mortality and morbidity rates have been losing significance [2]. Patient satisfaction is considered a tangible measure for evaluating the quality of health care [19,20]. It can also be considered the outcome of the health care system [20]. Moreover, there is research showing that better patient experiences are linked with lower hospital readmission rates, fewer complications, and lower mortality rates [1,13].

Various scales have been developed for measuring service quality in health care [2,21]. However, the SERVQUAL scale, which was created for measuring consumer perceptions of service quality in various domains [22], is the most used scale in the health care sector [2]. While most research on patient satisfaction is based on questionnaires, there is not much research based on the automatic analysis of UGC. Lee et al [23] conducted a study in which they compared the results obtained through text mining methods from user-generated data to the results obtained from surveys. They collected >50,000 tweets regarding the National Health Service in Great Britain, a public health service provider, and observed similar perceptions on service quality in health care when analyzing both social media content and traditional surveys.

### Statistics on COVID-19 Deaths in Finland and Andalusia

The number of COVID-19 deaths was chosen as a comparative metric between both countries as it provides a relatively clear outcome. It offers insight into the pandemic’s severity, but it should be interpreted with caution as we need to acknowledge that different countries adopted different approaches on the recording and reporting of deaths attributed to COVID-19. Moreover, these practices evolved over the course of the pandemic.

**Figure 1:**
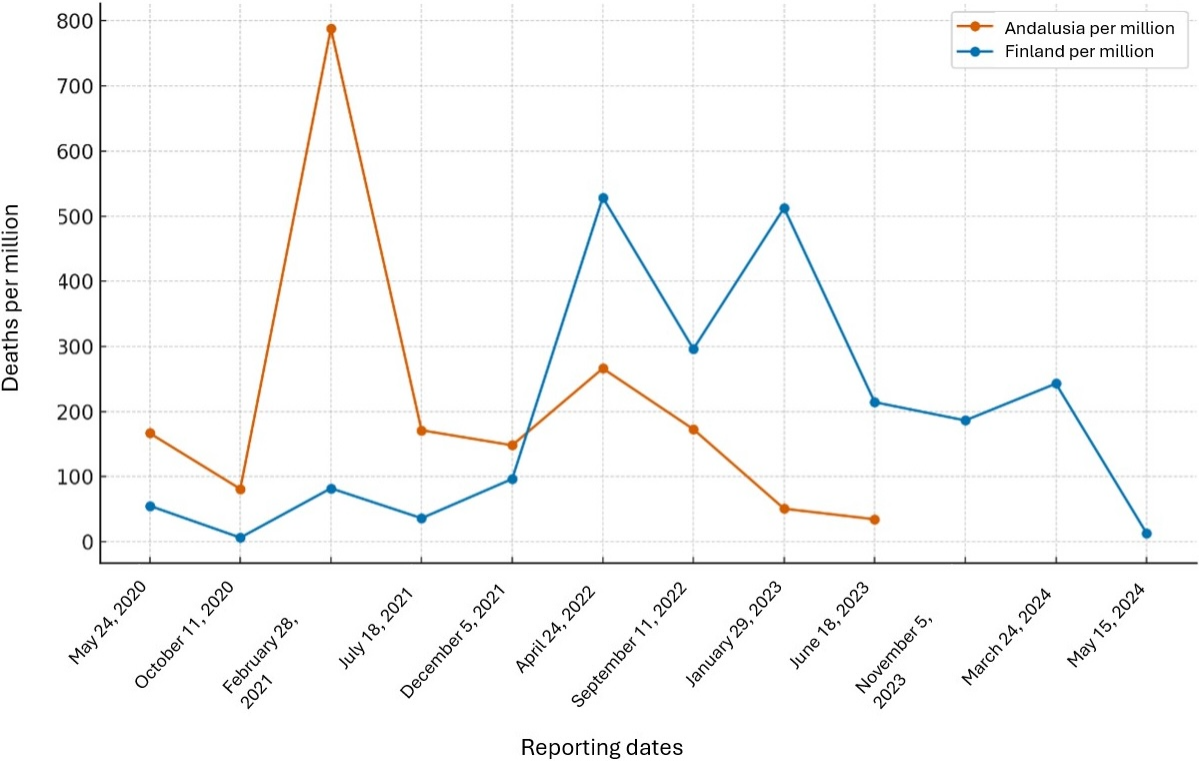
Number of deaths (per million people) in Andalusia and Finland across multiple time intervals.

Figure 1 shows the number of deaths per million over time for the 2 locations: Andalusia (in orange) and Finland (in blue). The x-axis represents the dates, whereas the y-axis represents deaths per million. Each data point represents approximately a 20-week period to offer insights with enough granularity into how the situation progressed over time in both locations. In the case of Finland, as COVID-19 data were available on the National Institute for Health and Welfare [24], the number of deaths was accounted for until the day of the last collection of Google Maps reviews (in May 2024). On the other hand, for Andalusia, data were included up to the point when they were no longer collected by the authorities (June 2023). In both cases, the start date of the data collection was January 1, 2020. To account for population size differences, the deaths per million people were calculated by dividing the absolute death numbers by the population of each location (8.5 million for Andalusia and 5.5 million for Finland).

In the case of Andalusia, the data regarding the number of deaths were collected from the National Center for Epidemiology [25,26]. Although the registration of new data stopped in July 2023, the existing data allowed for tracking the evolution of the pandemic in Andalusia.

In the case of Finland, the National Institute for Health and Welfare’s Finnish National Infectious Diseases Register reports ongoing key information on COVID-19.

As can be observed in Figure 1, both countries experienced multiple death peaks but with different temporal distributions. The number of deaths per million in Andalusia over the time frame reveals that this region experienced more severe and frequent spikes in mortality rates, especially in 2021. Finland shows lower peaks.

### Government Response to COVID-19 in Finland and Spain

To understand the similarities and differences in perceived service quality of primary health care, country-specific COVID-19 policies and their timing in Finland and Andalusia should also be considered. There are cross-country frameworks and datasets for measuring government response to COVID-19, such as the Oxford COVID-19 Government Response Tracker [27] and COVID-19 Health Systems Response Monitor [28,29]. They have been used in various cross-country analyses, such as comparing responses to COVID-19 [30]. There are also country- or region-specific frameworks such as the *severity index* for assessing nonpharmaceutical interventions on COVID-19 transmission in Spain [31]. These frameworks and indexes aggregate various response measures, including restrictions on public gatherings, school closures, curfews, and the maintenance of essential health services.

**Figure 2:**
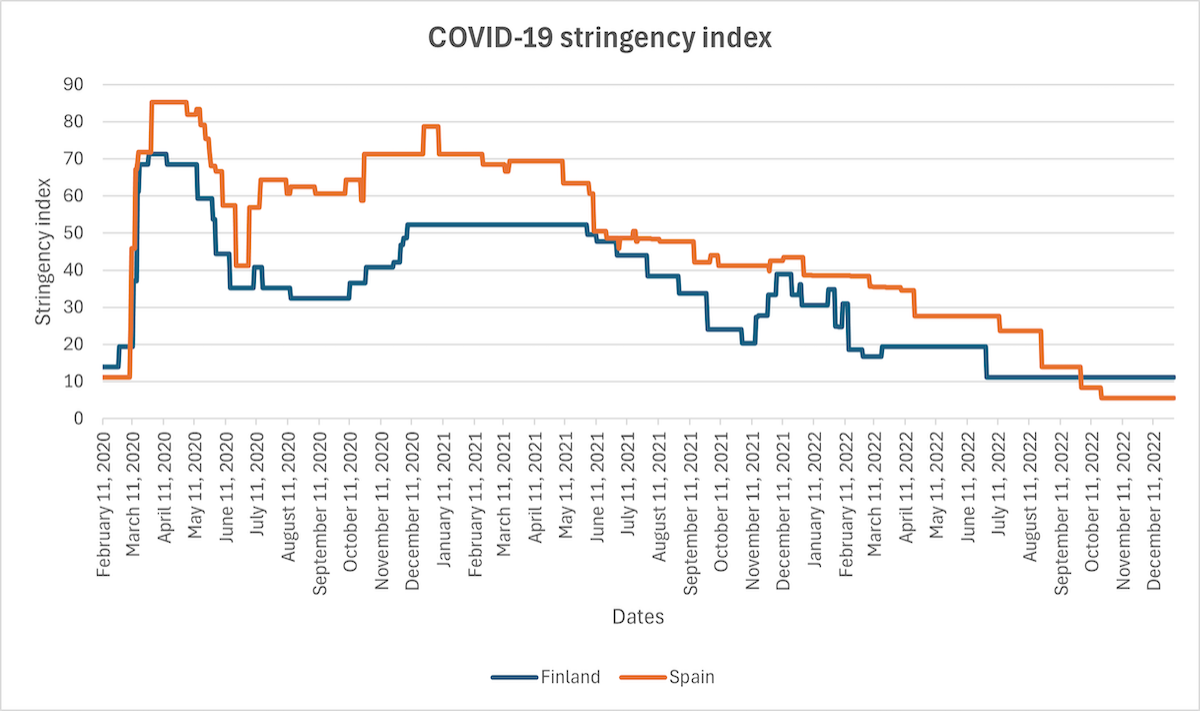
Comparison of the COVID-19 stringency index for Finland and Spain. Data source: Blavatnik School of Government, University of Oxford (2023).

To compare the policies in Finland and Andalusia, the COVID-19 stringency index suggested in the Oxford COVID-19 Government Response Tracker [32] may be used. It is a composite measure of 9 response metrics: school closures, workplace closures, cancellation of public events, restrictions on public gatherings, closures of public transport, stay-at-home requirements, public information campaigns, restrictions on internal movements, and international travel controls. The data for the stringency index were collected at the national level for Finland and Spain [33] from January 1, 2020, to December 31, 2022, and they are shown in Figure 2. The figure shows that the response measures were stricter in Spain almost throughout the COVID-19 pandemic, with an exception at the end starting from November 2022.

## Methods

### Data Collection

#### Overview

**Figure 3:**
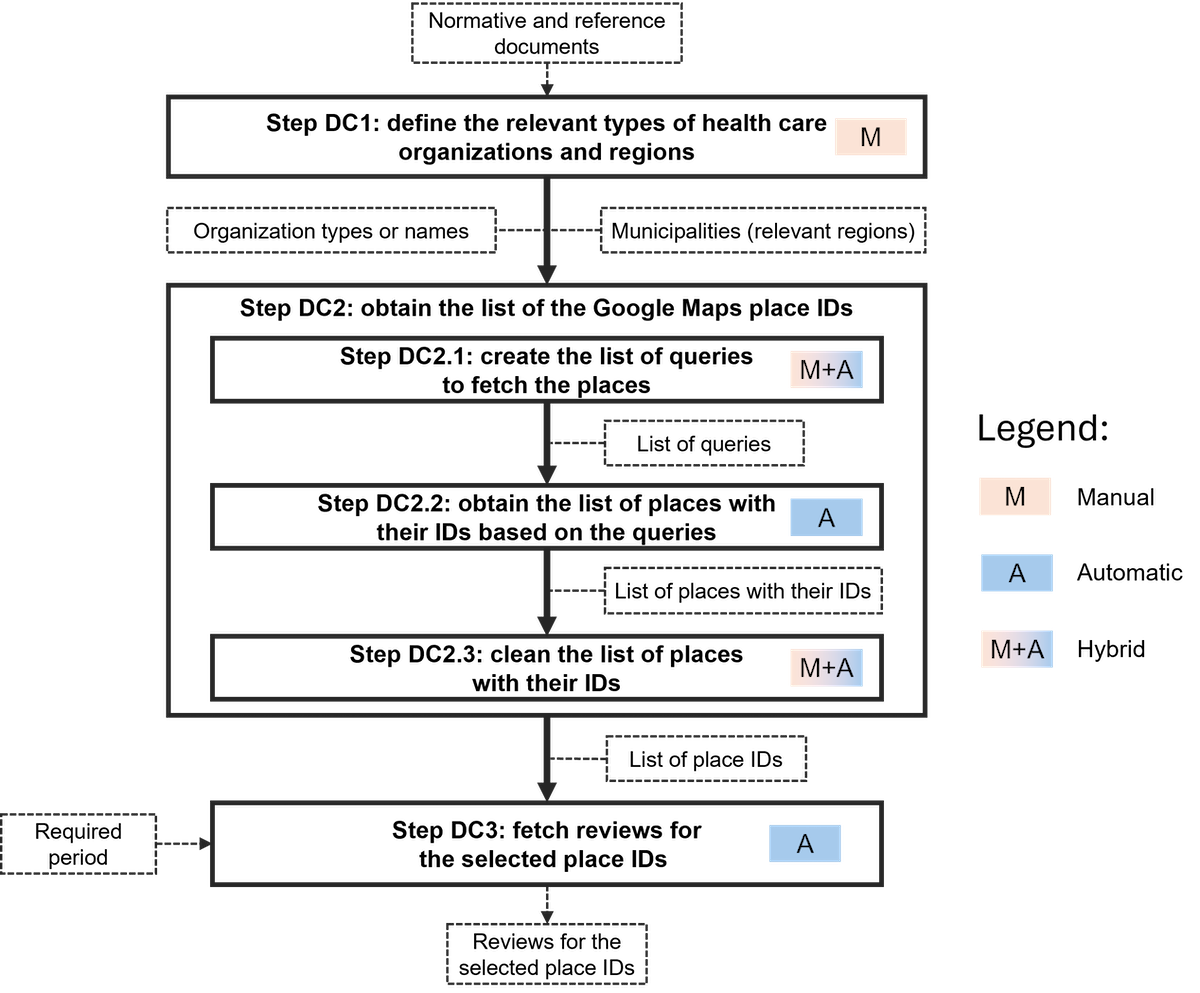
The process of data collection.

Google Maps reviews for primary health care organization locations in Finland and Andalusia, Spain, were collected for this study. The data collection process involved three main steps (Figure 3):

1. Defining the types of organizations, which represent primary health care providers in the target regions (Finland and Andalusia in our case)
2. Obtaining the list of Google Maps place IDs (unique representations of relevant organizations in Google Maps) for the selected types of organizations in the target regions
3. Fetching reviews for the selected place IDs

Figure 3 also highlights which steps were manual, automatic, or hybrid. The Outscraper [34] service was used for fetching reviews. The need for using such a service was justified by the limitations of the publicly available Google Maps application programming interface, which allows only 5 reviews to be fetched for 1 place.

The first step of data collection (ie, defining the types of organizations that represent primary health care providers [step DC1 in Figure 3]) was based on manual analysis of country-specific normative documents such as national and regional laws, decrees, and acts. This analysis implied the identification of the meaning of the term *primary health care* for Finland and Andalusia, which helped identify relevant types of health care organizations—primary health care providers. To make further analysis correct and comparable, we also aligned the lists of organization types for both countries. Dental care was excluded from the analysis.

In Andalusia, primary health care includes “preventive, curative and rehabilitative care as well as health promotion, health education and environmental health surveillance” [35]. According to article 5 of the Andalusian Department of health [36], there are 3 types of primary care centers in Andalusia: primary health care centers (*centros de salud*), local clinics (*consultorios locales*), and auxiliary clinics (*consultorios auxiliares*).

In Finland, public health services are divided into primary and specialized health care [37]. According to the Finnish Health Care Act 1326/2010, primary health care means “public health services provided by local authorities, health promotion, and any related provision of health counseling and health checks, oral health care, medical rehabilitation, occupational health care, environmental health care, as well as emergency medical care, outpatient care, home nursing, at-home hospital care and inpatient care, mental health services, and substance abuse services where these are not covered by social services or specialized medical care.” Although various organizations are involved in the provision of primary health care services, the main primary health care service providers are the following: health centers (*terveyskeskus*), health stations (*terveysasema*) and maternity and child health clinics (*neuvola*), and welfare centers (*hyvinvointikeskus*) in some cases [37,38].

In addition to the list of the relevant types of health care organizations, a list of relevant locations was needed. To this aim, lists of municipalities for Andalusia and Finland were defined. These lists of municipalities could be obtained either from the descriptions of medical regions (which can be named differently in each country, eg, *welfare regions* in Finland) or from the official list of the countries’ municipalities. It was possible to obtain a list of health centers (*centros de salud*), local clinics (*consultorios locales*), and auxiliary clinics (*consultorios auxiliares*) for Andalusia via a regional service [39]. This list also specifies the municipality and province for each health center. The list of the Finnish municipalities was taken from the Statistics Finland website [40].

The second step in the data collection process was aimed at fetching the Google Maps place IDs for the health care organizations needed (step DC2 in Figure 3).

The general process for this step and its peculiarities for Andalusia and Finland are described in the following sections.

#### Creating the List of Queries to Fetch the Places (Step DC2.1)

The primary approach was to concatenate the needed organization types with municipalities. For example, *terveyskeskus* as the needed organization type was concatenated with Helsinki, Espoo, and Tampere, among others (municipalities). We tried creating queries without municipalities (only the organization type and country filter or the organization type plus the medical region), but the resulting list of places was shorter.

Google Maps and Outscraper understand the meaning of words in the query and provide not only places with exact matches but also synonymous places. In some cases, it was useful for data collection purposes, for example, when the name of the organization had been changed, whereas in some other cases, it provided excessive query results, and it was one of the reasons for additional data cleaning. For example, we did not include *hyvinvointikeskus* (welfare center) as an organization type in the queries because we primarily obtained relevant *hyvinvointikeskus* via queries for *terveyskeskus* and *terveysasema*. The problem with this term was that it is very general and generated a lot of irrelevant results (beyond primary health care). We left out outside locations such as schools even though part of primary health care for children and adolescents is organized in such locations. However, including schools would have resulted in a set of ratings and reviews in which most would have been irrelevant. Occupational health is largely organized through private health centers, which were left out from data collection.

#### Obtaining a List of Places Based on the Queries (Step DC2.2)

A Python script (Python Software Foundation) was created for fetching a list of Google Maps places. This step was the same for Andalusia and Finland; the only difference was in the country filter (Spain or Finland). The following data were collected: *place_name*, *place_id*, *place_type*, *place_category*, address, municipality, postal code, country, number of reviews, rating, and *place_location_link*.

#### Data Cleaning (Automatic, Semiautomatic, and Manual; Step DC2.3)

This phase included the automatic dropping of duplicate place IDs and places without ratings or reviews. In addition, as mentioned previously in the description of step DC2.2, several irrelevant places were obtained through the Outscraper queries. Thus, semiautomatic and manual analysis and exclusion of irrelevant places were needed.

For Andalusia, we excluded the following places semiautomatically: places outside Andalusia based on the address column (eg, big cities in Spain, such as Madrid and Valencia), places of irrelevant types via a filter using the *place_type* column (relevant *place_types* were manually selected from the list of *place_types*—unique values), and places with *Hospital* in the name column (for relevant types only). Places with >30 reviews were manually checked. For Finland, we excluded dental health care centers (*hammashoitola*), hospitals (*sairaala*), private health centers (eg, *terveystalo*), and some other irrelevant organization types.

The suggested approach has a risk of including irrelevant places and excluding relevant ones. However, it can be minimized via careful analysis and selection of place types and categories on Google Maps (as we did).

#### Fetching Reviews for the Selected Place IDs (Step DC3)

A script was created to fetch the needed reviews, which took the list of place IDs and the required period as input. The review texts were collected in the original language. The list of the required place IDs was created in the previous step (DC2). To define the period for collecting reviews, we considered the dates regarding COVID-19 provided by the World Health Organization (WHO). The director-general of the WHO declared the COVID-19 outbreak a public health emergency of international concern on January 30, 2020. On March 11, 2020, the WHO declared COVID-19 a pandemic. On May 5, 2023, the WHO officially declared the end of COVID-19 as a public health emergency. Regardless of the common events identified worldwide, each country and region had specific timelines regarding the pandemic. Thus, we decided to collect reviews several years before January 30, 2020, and covering the COVID-19 pandemic period and the period after. Thus, the reviews were collected from January 1, 2013, to May 14, 2024. Table 1 shows the numbers of fetched reviews in Andalusia and Finland.

**Table 1:** Overview of the collected data.

	Andalusia	Finland
Reviews, n	42,796	12,247
Reviews with text, n/N (%)	27,756/42,796 (64.86)	6558/12,247 (53.55)
Organizations, n	1016	604
Period	January 1, 2013-May 14, 2024	January 1, 2013-May 14, 2024
Reviews with text, mean (SD)	27.29 (46.68)	10.87 (20.39)
Reviews per organization, range	0-328	0-228
Reviews at quartile 1	1	1
Reviews at mean	5	4
Reviews at quartile 3	32	12

### Results of Data Collection

The resulting table for review data collection had the following structure: *place_id*, *place_name*, *place_location_link*, *review_datetime_utc*, *review_timestamp*, *review_rating* (number of stars on Google Maps), *review_likes*, *review_text*, *review_link*, and region. Table 2 shows an example of a review for one of the medical centers in Finland. This review was posted on September 15, 2023, at 6:48 PM. The user’s rating of the review was 5. Most of the review texts from Finland were in Finnish, whereas for Andalusia, they were in Spanish; however, part of the reviews were in other languages. The organizations with the highest number of reviews with text in Finland were Helsinki University Hospital Myllypuro Laboratory (n=153 reviews), Kontinkangas Health and Well-being Centre (n=228), and Kalasatama Health and Well-being Centre (n=199), whereas in Andalusia, they were the Limonar Health Center (n=328), Dr Joaquín Pece Health Center (n=281), and Las Lagunas Health Center (n=283).

**Table 2:** An example of the data from a fetched review.

Variable	Description
place_id	ChIJjxAy8d4JkkYRrXCQ_fJyEow
place_name	Health center name X
place_location_link	A Google Maps location URL
review_datetime_utc	September 15, 2023, at 6:48 PM
review_rating	5
review_text (translated and anonymized)	Review text
review_link	A Google Maps location URL
Region	Finland

### Data Analysis Process

The data extracted from Google Maps using Outscraper were stored in a tabular format. First, we filtered out the non–Spanish-language text reviews for Andalusia and the non–Finnish-language text reviews for Finland using *langdetect* [24]. A total of 7.9% (2195/27,756) non–Spanish-language reviews and 13.9% (913/6558) non–Finnish-language reviews were removed, resulting in 25,561 reviews for Andalusia and 5645 reviews for Finland.

Initially, to conduct the sentiment analysis, 2 tools were considered: *pysentimiento* [26] and AFINN [41]. *pysentimiento* is a Python toolkit; the model is transformer based; and it can analyze text in Spanish, English, Italian, or Portuguese. To add a new language to this toolkit, it is necessary to train a new model. AFINN [42] is a wordlist-based approach to sentiment analysis that uses preannotated dictionaries where each word is assigned a sentiment score between –5 and 5. A score of 0 means a neutral sentiment, a negative score means a negative sentiment, and a positive score means a positive sentiment. The AFINN tool produces as output the sum of the scores of the emotionally loaded words in the text. Thus, for example, a long sentence with many negatively loaded words will receive a lower score than a short sentence with proportionally as many negative words. This scoring reflects the fact that, if a user makes the effort to write a long and negative review, it is given more importance than a short review with proportionally as many negative words.

As a disclaimer for scientific reproducibility, at the time this research was being conducted, the version available on the Python Package Index repository did not include the Finnish language, but the one on the official GitHub repository did. Furthermore, AFINN offers a Spanish dictionary available on GitHub.

Finally, *pysentimiento* was discarded for 2 primary reasons: first, the analysis could not be conducted for the Finnish language, and second, its output format was incompatible with that of the AFINN tool.

Deep learning–based methods were applied for confirming the results obtained through AFINN. The following fine-tuned deep transfer learning models were used: bert-finnish-sentiment-analysis-v2 [43] for Finnish and twitter-XLM-roBERTa-emotion-es [44,45] for Spanish. The Finnish sentiment analyzer is a fine-tuned version of the uncased bidirectional encoder representations from transformers deep transfer learning model for Finnish [46]. The fine-tuning was conducted using the nisancoskun/finnish_sentiment_data dataset [47]. The sentiment analyzer classified the reviews into positive and negative. The Spanish sentiment analyzer was based on the multilingual XLM-roBERTa-base model [48] that has been fine-tuned for emotion classification in Spanish. It classified the reviews into the following 7 emotions: anger, disgust, fear, joy, sadness, surprise, and other, the latter representing other emotions or the absence of any emotion. In our analysis, we regarded the emotions anger, disgust, fear, and sadness as negative and joy and surprise as positive.

The entire dataset was organized into a DataFrame and subsequently divided into 3 distinct subsets: one representing the period before the COVID-19 pandemic, one representing the period during the pandemic, and one representing the period after the end of the pandemic had been declared [49]. This was the first step of our pipeline, as can be seen in step DC1 in Figure 3. Table 3 shows the date ranges of the DataFrames.

In this context, the decision to divide the data into 3 temporal phases (before, during, and after the pandemic) not only responds to an obvious chronological logic but is also widely justified from a methodological, social, and health care perspective. This segmentation allows for a more accurate capture of variations in public perceptions of the health care system, as well as changes in the nature of interactions among patients, health care professionals, and institutions.

**Table 3:** Date ranges of the periods before, during, and after the COVID-19 pandemic.

Period	Beginning	End
Before the pandemic	January 1, 2013	March 10, 2020
During the pandemic	March 11, 2020	May 4, 2023
After the pandemic	May 5, 2023	May 15, 2024

Before the pandemic, the health care system operated under a relatively stable paradigm. In-person consultations were the norm, and user ratings tended to focus on classic aspects such as waiting times, quality of care received, and administrative efficiency. During this period, sentiment analysis reveals a more neutral or predictable emotional distribution influenced by structural or regional factors but without major external changes that distort general perceptions.

The outbreak of the pandemic caused a profound disruption in the organization and functioning of health care systems worldwide. During this phase, there was an unprecedented overload of hospital services, as well as the suspension or postponement of numerous nonurgent consultations and procedures. Furthermore, the use of alternative care channels such as telemedicine, which until then had been marginally used, became widespread. Consequently, the emotional content of opinions on and assessments of medical care during this period is qualitatively different. Fear, uncertainty, frustration over the lack of resources, and gratitude toward health care professionals emerge as dominant emotions, making it essential to analyze this period separately to avoid distortions in the analysis models.

The period after the pandemic, while not implying a full return to prepandemic conditions, marks a new scenario characterized by a re-evaluation of the lessons learned from the health care system. The population expresses new expectations regarding the quality and accessibility of services, as well as a higher level of demands on institutional management. Similarly, new topics emerge in patient reviews and opinions, such as the aftereffects of post–COVID-19 condition, the impact on mental health, and the accumulation of waiting lists. This phase also allows us to observe how the social perception of the health care system underwent a lasting transformation, reflecting a heightened awareness of its vulnerability and the need for resilience.

From a methodological perspective, temporal segmentation is essential for correctly interpreting the data. The meaning of certain expressions changes radically depending on the context. For example, a statement such as *I was not seen in the emergency room* can have very different implications if it is uttered before the pandemic, during the hospital collapse, or during the recovery phase. Similarly, the volume of reviews or publications may have skyrocketed during the pandemic period driven by the emotional burden and visibility of the health care system, requiring differentiated statistical treatment.

Finally, this temporal division offers added value from the perspective of health care management and public policy. It allows for clearly identifying critical moments in the system, evaluating the effectiveness of the measures adopted, and drawing lessons applicable to future crises. Furthermore, it facilitates the design of more effective communication strategies aligned with the emotions and expectations of the population at each phase.

Online reviews reflect patient experience with certain delay. However, most reviews are written within the first week of the patient experience: “Nearly half (47%) of patients are most likely to leave a review within 24 hours of their appointment, and 74% said they would be more inclined to do so if their provider asked them to” [50]. Thus, as the periods for analyzing sentiment trends in our research were long (more than a year) and most reviews are written within a week, the influence of this delay was insignificant for this analysis and was not considered when we created the subsets.

**Figure 4:**
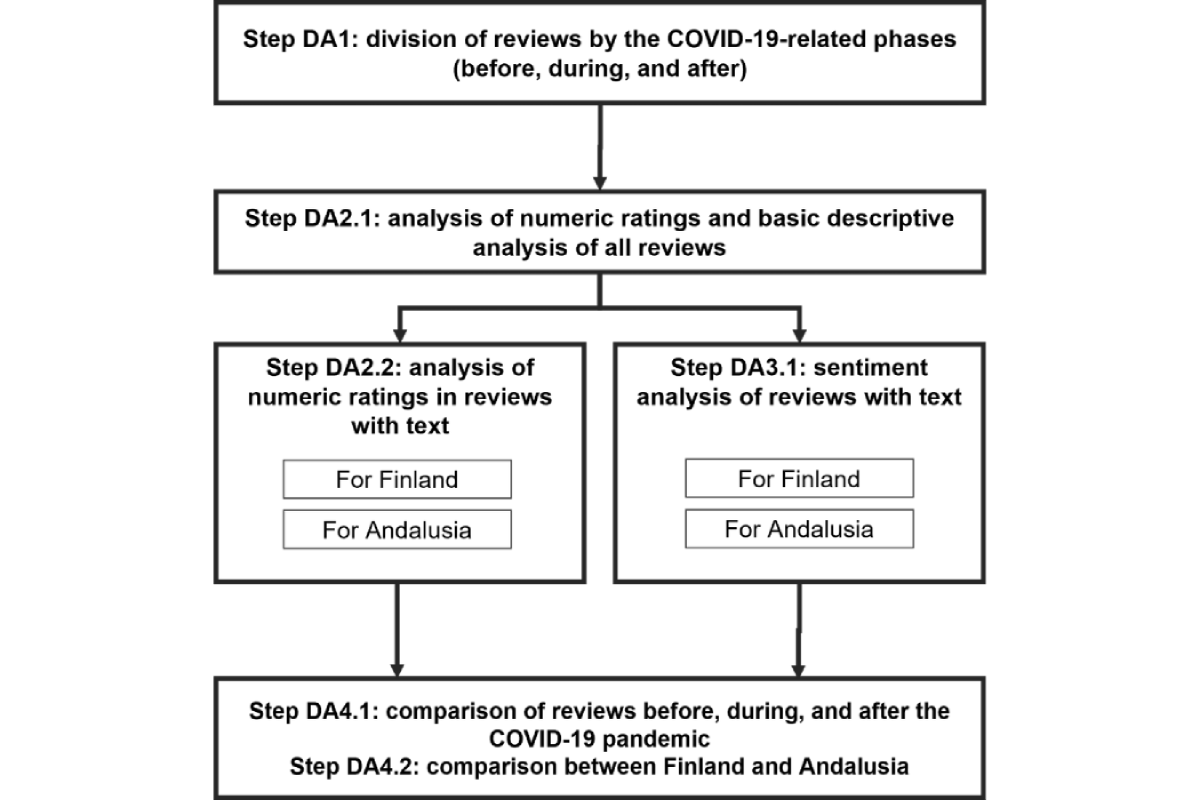
The process of data analysis.

After the datasets had been selected, the UGC (Google Maps reviews) was analyzed (step DA2.1 in Figure 4). Analysis of the most frequent words and nouns associated with positive and negative review sentiment was also conducted. A simple categorization of the reviews was proposed dividing positive and negative ones: reviews above and including a rating of 4 stars were considered positive, whereas reviews below and including a rating of 2 stars were understood as negative. Language-specific text processing steps were applied to the data. For this purpose, we used natural language processing models from the Python spaCy library [51]. The pipeline involved tokenization, lemmatization, stop word removal, and filtering of punctuation. The most common words and nouns were extracted from the processed tokens. After this first step, we removed reviews without text, and we conducted the same analysis (step DA2.2). Figure 4 shows all the steps taken in the processing of the data.

### Ethical Considerations

Following the guidelines of the ethics committee of Haaga-Helia University of Applied Sciences, an institutional ethical review was deemed unnecessary for this study, which collected and analyzed publicly available social media data. All presented reviews were translated into English and anonymized to avoid association with any particular user of the Google Maps platform.

## Results

The results of the data analysis are presented in the same order as the phases of the process shown in Figure 4. We will first describe the results for Finland and, thereafter, for Andalusia.

### Results for Finland

**Figure 5:**
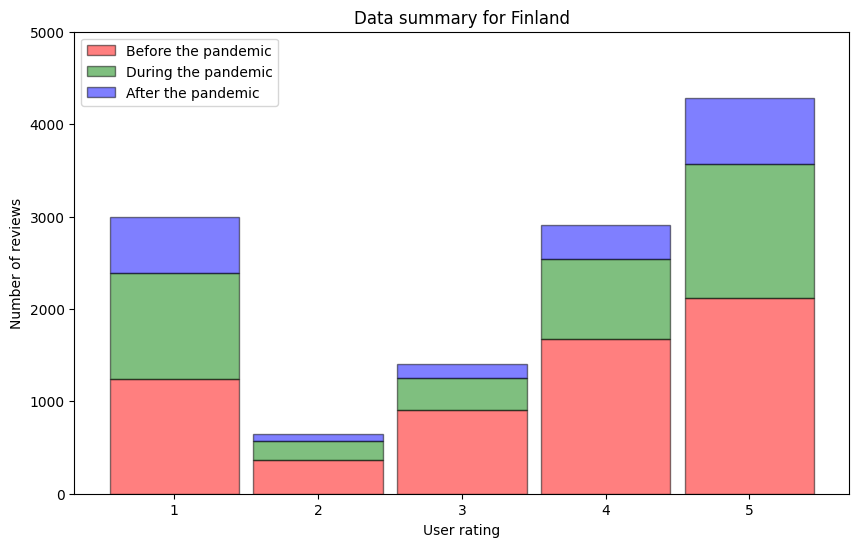
Numeric user ratings before, during, and after the pandemic in Finland.

As described in the previous section, the first analysis captured the frequency distribution of user ratings (step DA2.1 in Figure 4). Google Maps ratings range across a scale from 1 to 5. Figure 5 presents the aggregated data from Finland across the defined phases—before the pandemic, during the pandemic, and after the pandemic—and the cumulative data from all 3 periods. From the prepandemic period, a total of 6294 reviews were analyzed, with a mean rating of 3.48 (SD 1.489) and a median rating of 4.0 (IQR 3), which indicates a generally positive user sentiment. During the pandemic, 4026 reviews were processed, with a mean rating of 3.31 (SD 1.661) and a median rating of 4.0 (IQR 4), reflecting a similar trend in user feedback. From the postpandemic period, we analyzed 1932 reviews, with a mean rating of 3.28 (SD 1.826) and a median rating of 4.0 (IQR 4; Table 4).

**Table 4:** Summary of the numeric ratings of all reviews extracted for Finland.

		Before the pandemic (n=6294)	During the pandemic (n=4026)	After the pandemic (n=1932)
**Review numeric rating, n (%)**				
	5	2114 (33.59)	1452 (36.07)	722 (37.37)
	4	1670 (26.53)	868 (21.56)	375 (19.41)
	3	908 (14.43)	344 (8.54)	157 (8.13)
	2	362 (5.75)	209 (5.19)	78 (4.04)
	1	1240 (19.7)	1153 (28.64)	600 (31.06)
Rating, median (IQR)		4 (3)	4 (4)	4 (4)
Rating, mean (SD)		3.48 (1.489)	3.31 (1.661)	3.28 (1.826)

Figure 5 allows us to observe that the plots’ shapes have the same form. The values for the median and mean ratings in Table 4 confirm what can be visually observed in the figure—the numeric user ratings did not show noteworthy changes in Finland. In Table 4, we observe that the median stayed the same and the mean showed a very slight decrease.

**Figure 6:**
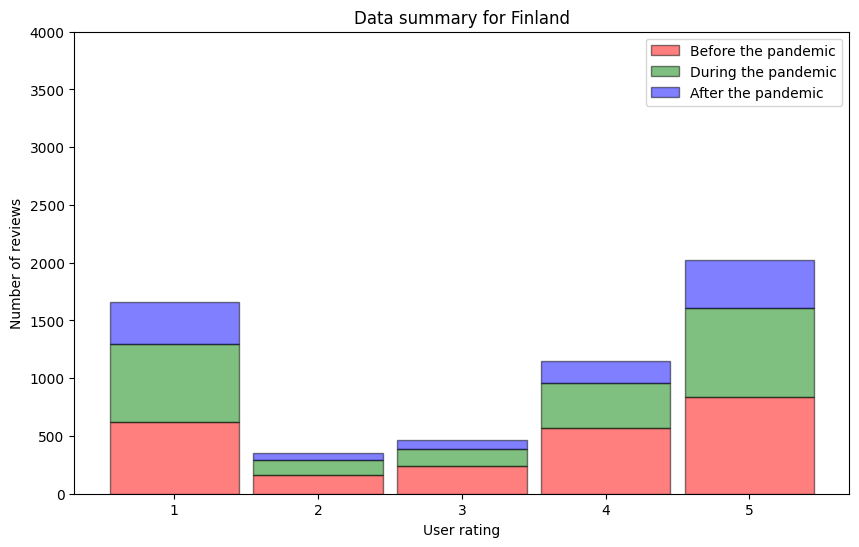
Numeric user ratings before, during, and after the pandemic in Finland including only reviews with text.

Before conducting the sentiment analysis on the textual reviews, we took out the reviews without written text or not written in Finnish or Spanish (step DA2.2). The data regarding the Finnish reviews with text are shown in Figure 6. In the figure, we included the information from before the pandemic, 2423 reviews, where the median rating was 4.0 (IQR 4) and the mean rating was 3.34. During the pandemic, we processed 2119 reviews with a median rating of 4.0 (IQR 4) and a mean rating of 3.22. Finally, data from the period after the pandemic consisted of 1103 reviews with a median rating of 4.0 (IQR 4) and a mean rating of 3.21. The numeric data can be found in Table 5. These figures were calculated to ensure that there is no noteworthy difference in the numeric ratings of the reviews with text and those without text. As no noteworthy difference was found, we could proceed to the sentiment analysis of the reviews with text.

**Table 5:** User ratings for Finland (only reviews with text).

		Before the pandemic (n=2423)	During the pandemic (n=2119)	After the pandemic (n=1103)
**Review numeric rating, n (%)**				
	5	833 (34.38)	778 (36.72)	411 (37.26)
	4	568 (23.44)	389 (18.36)	197 (17.86)
	3	238 (9.82)	147 (6.94)	78 (7.07)
	2	164 (6.77)	132 (6.23)	53 (4.81)
	1	620 (25.59)	673 (31.76)	364 (33)
Rating, median (IQR)		4 (4)	4 (4)	4 (4)
Rating, mean (SD)		3.34 (2.58)	3.22 (2.94)	3.21 (2.98)

**Figure 7:**
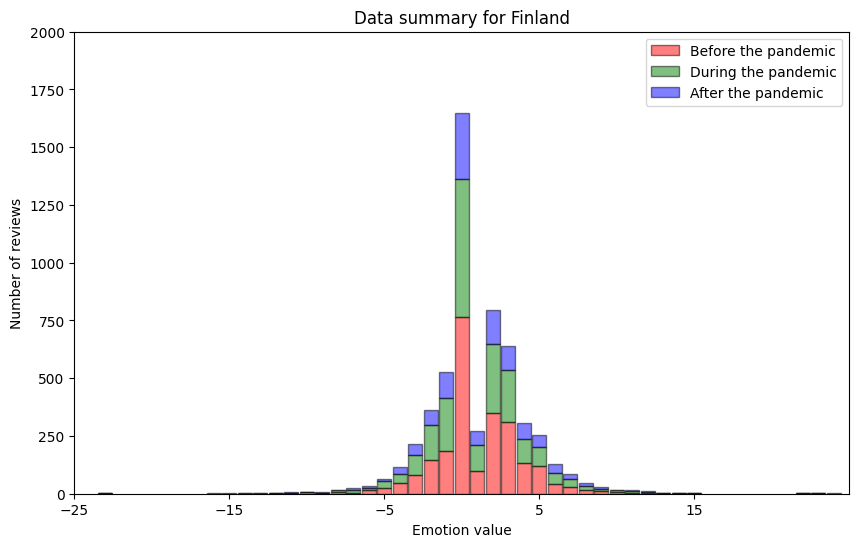
Results of sentiment analysis for Finland using AFINN.

Finally, we processed the reviews using the AFINN tool (step DA3.1). The results are shown in Figure 7 and Table 6. Figure 6 shows stacked information on these results. The minimum sentiment value obtained using the AFINN tool was −23 (most negative), and the maximum value obtained was 23 (most positive). The median was 0.0, and the mean was 1.01. During the pandemic, the minimum sentiment value was −14, and the maximum value was 15, with a median of 0.0 and a mean of 0.85. After the pandemic, we found a minimum sentiment value of −16 and a maximum value of 24, with a median of 0.0 and a mean of 1.03.

**Table 6:** Sentiment analysis result summary for Finland (N=6363).

	Reviews, n (%)	Sentiment value, median (IQR)	Sentiment value, mean (SD; range)
Before the pandemic	2423 (38.08)	0.0 (3)	1.01 (2.96; –23 to 23)
During the pandemic	2119 (33.3)	0.0 (4)	0.85 (2.99; –14 to 15)
After the pandemic	1103 (17.33)	0.0 (4)	1.03 (3.35; –16 to 24)

**Figure 8:**
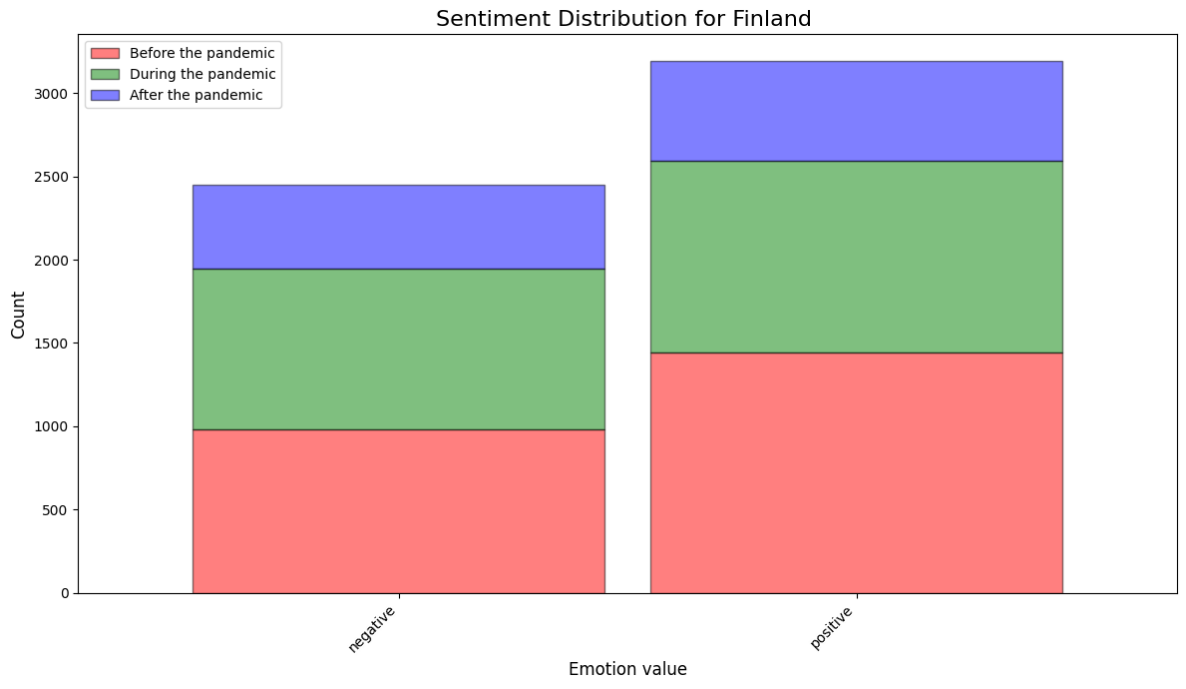
Results of sentiment analysis using the deep learning–based model for Finland.

Figure 8 shows the results of the sentiment analysis using the deep learning–based method. Similarly to the lexicon-based method, this method also showed that the distribution of the positive and negative sentiment in Finnish-language reviews stayed relatively stable for the periods before, during, and after the pandemic.

The numerical data obtained are shown in Table 7. As can be observed, there were more positive than negative emotions in the reviews

**Table 7:** Summary of emotions obtained for Finnish-language reviews using the deep learning–based model. The score for positive reviews was 1, and the score for negative reviews was −1.

	Positive reviews, n	Negative reviews, n	Sentiment score, mean (SD)	Sentiment score, median (IQR)
Before the pandemic	1442	980	0.19 (0.96)	1 (2)
During the pandemic	1151	967	0.09 (1.00)	1 (2)
After the pandemic	600	502	0.09 (1.00)	1 (2)

### Results for Andalusia

**Figure 9:**
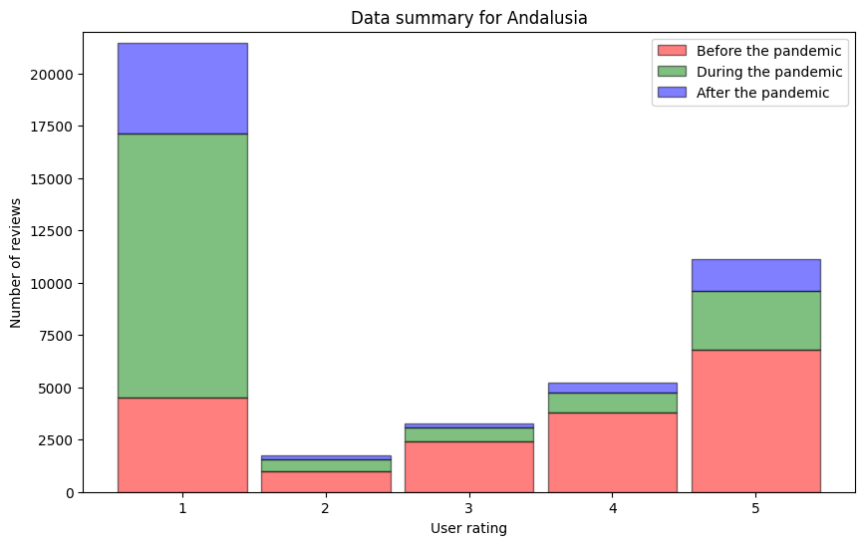
Numeric user ratings before, during, and after the pandemic in Andalusia.

In the following paragraphs, we present the same analysis for the data from Andalusia as that presented for the data from Finland in the previous section. First, we present the information about the numeric ratings of all reviews (step DA2.1 in Figure 4). The data are shown in Figure 9 and Table 8. As can be observed, contrary to the data for Finland, the ratings changed. The data before the pandemic showed similar ratings as those in the data from Finland, with the median being 4.0 (IQR 4) and the mean being 3.40. During the pandemic, ratings worsened in Andalusia, with the median being 1.0 (IQR 2) and the mean being 1.91. Finally, after the pandemic, the median rating continued to be 1.0 (IQR 3), and the mean rating was 1.99.

**Table 8:** Summary of the numeric ratings of all reviews extracted for Andalusia.

		Before the pandemic (n=18,506)	During the pandemic (n=17,592)	After the pandemic (n=6739)
**Review numeric rating, n (%)**				
	5	6819 (36.85)	2796 (15.89)	1524 (22.61)
	4	3778 (20.42)	983 (5.59)	447 (6.63)
	3	2398 (12.96)	665 (3.78)	230 (3.41)
	2	999 (5.4)	554 (3.15)	192 (2.85)
	1	4512 (24.38)	12,594 (71.59)	4346 (64.49)
Rating, median (IQR)		4 (4)	1 (2)	1 (3)
Rating, mean (SD)		3.39 (2.50)	1.91 (2.40)	2.20 (2.94)

**Figure 10:**
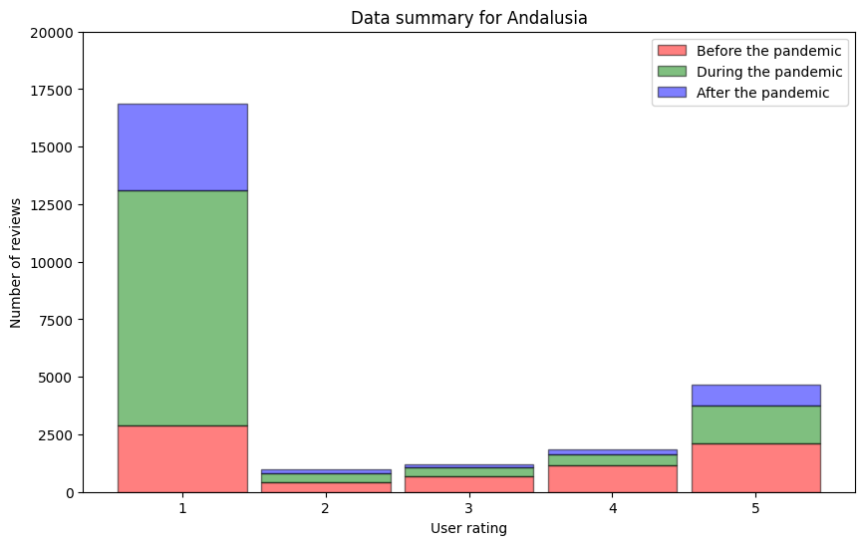
Numeric user ratings before, during, and after the pandemic in Andalusia including only reviews with text.

After this analysis, we took out all reviews without text and in a language different from Spanish. We show the information for the remaining reviews in Figure 10 and Table 9 (step DA2.2). For the period before the pandemic, we found 7281 reviews, in which the median rating was 3.0 (IQR 3) and the mean rating was 2.88. During the pandemic, negative reviews became more frequent. In this period, we found 13,098 reviews, with a median rating of 1 (IQR 0) and a mean rating of 1.69. Finally, after the pandemic, the data followed the same tendency. We processed 5182 reviews, with a median rating of 1.0 (IQR 1) and a mean rating of 1.91.

**Table 9:** Summary of ratings of the reviews with text in Andalusia.

			Before the pandemic (n=7281)		During the pandemic (n=13,098)		After the pandemic (n=5182)
**Review numeric rating, n (%)**							
	5	2106 (28.92)		1638 (12.51)		929 (17.93)	
	4	1157 (15.89)		475 (3.63)		207 (3.99)	
	3	688 (9.45)		390 (2.98)		128 (2.47)	
	2	424 (5.82)		406 (3.1)		142 (2.74)	
	1	2906 (39.91)		10,189 (77.79)		3776 (72.87)	
Rating, median (IQR)			3 (4)		1 (0)		1 (1)
Rating, mean (SD)			2.88 (2.96)		1.69 (1.99)		1.91 (2.52)

**Figure 11:**
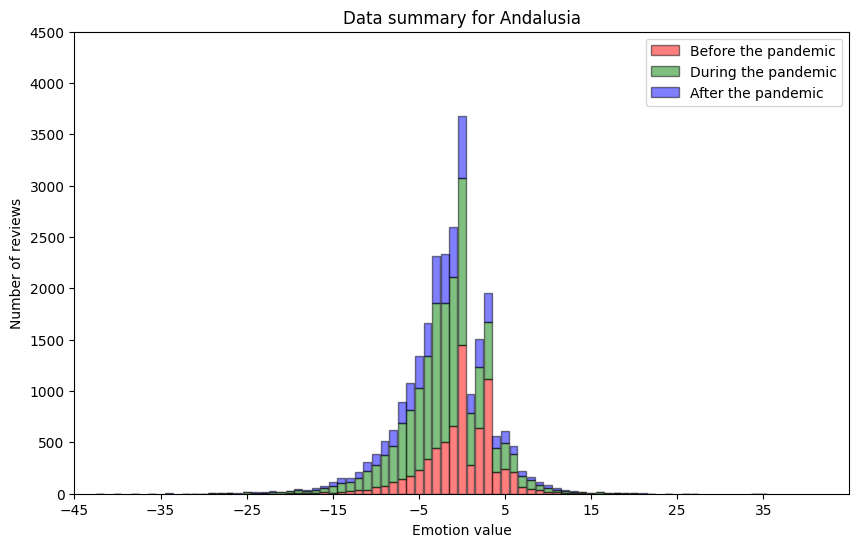
Results of sentiment analysis for Andalusia using AFINN.

As we could expect with these data from Andalusia, the sentiment analysis showed similar results as those for the ratings (step DA3.1). These results are shown in Figure 11 and Table 10. For the period before the pandemic, we processed 8225 reviews, for which we found a minimum sentiment value of −42 and a maximum value of 22. The median was 0.0, and the mean was −0.26. For the period during the pandemic, we retrieved 13,972 reviews, with the minimum sentiment value being −36 and the maximum value being 26.0. The median was −2.0, and the mean was −2.31. For the period after the pandemic, in the 4735 reviews retrieved, the minimum sentiment value was −44.0, and the maximum value was 35.0. The median was −2.0, and the mean was −2.38.

**Figure 12:**
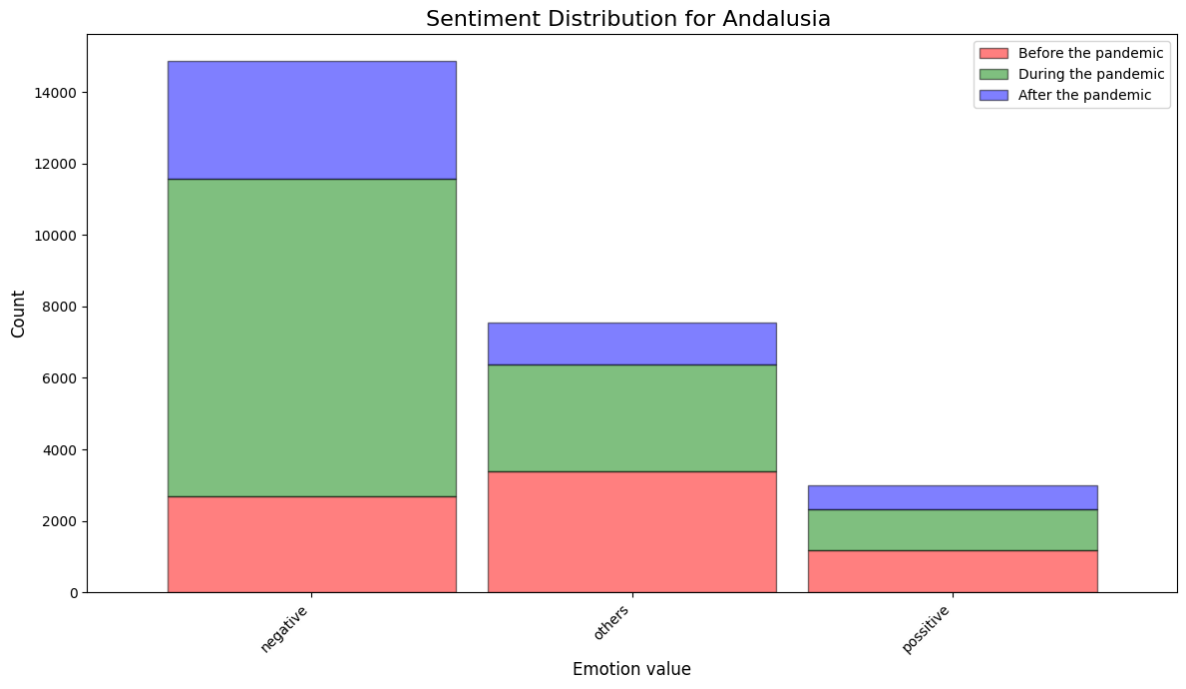
Sentiment analysis results obtained using the deep learning–based model for Andalusia.

Figure 12 and Table 11 show the results of the sentiment analysis using deep learning–based methods for Andalusia. The results obtained were in line with the previous results obtained using the lexicon-based sentiment analysis tool. The data demonstrate a preponderance of negative emotions over positive emotions. A substantial proportion of the data are attributable to multiclass classification.

**Table 10:** Sentiment analysis result summary for Andalusia using AFINN (N=25,561).

	Reviews, n (%)	Sentiment value, median (IQR)	Sentiment value, mean (SD; range)
Before the pandemic	7281 (28.48)	0.0 (0.055)	−0.26 (0.048; –42 to 22)
During the pandemic	13,098 (51.24)	−2.0 (0.055)	−2.31 (0.055; –36 to 26)
After the pandemic	5182 (20.27)	−2.0 (0.055)	−2.39 (0.063; –44 to 35)

**Table 11:** Summary of sentiment obtained for Andalusia using deep learning–based methods. Multiclass classification was used, and scoring was as follows: positive=1, negative=−1, and other=0.

	Positive reviews, n	Negative reviews, n	Reviews with “other” sentiment, n	Sentiment score, mean (SD)	Sentiment score, median (IQR)
Before the pandemic	1190	2697	3382	−0.39 (0.724)	0 (1)
During the pandemic	1149	8874	2983	−0.77 (0.670)	−1 (1)
After the pandemic	648	3298	1174	−0.67 (0.620)	−1 (1)

### Lexical Analysis of the Reviews

**Figure 13:**
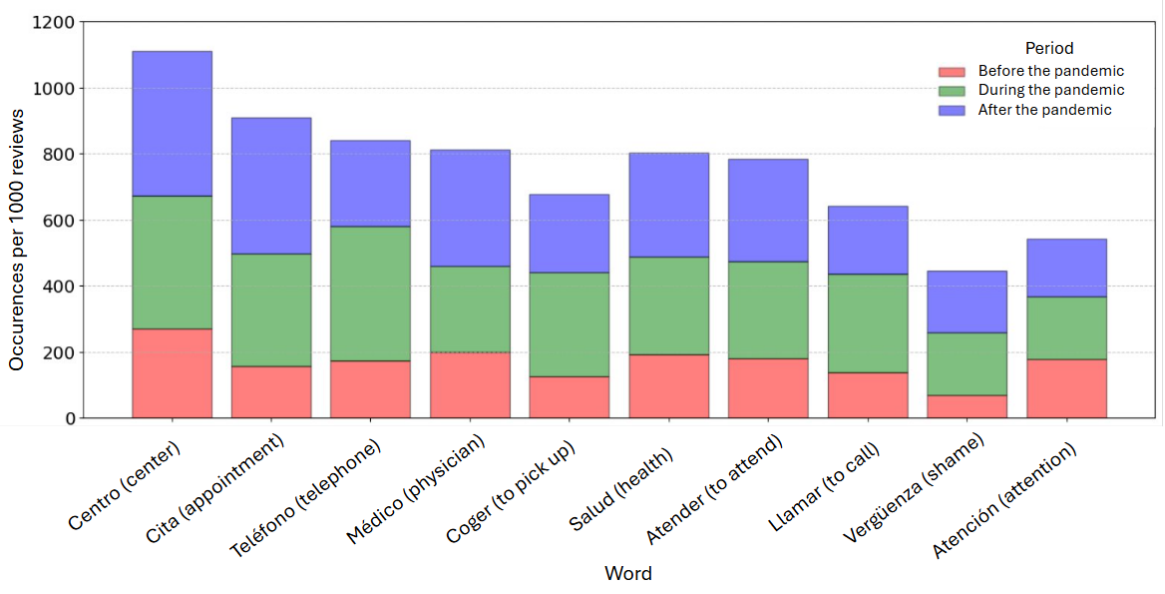
Relative frequency of the top words in Spanish-language reviews (per 1000 reviews) before, during, and after the pandemic.

**Figure 14:**
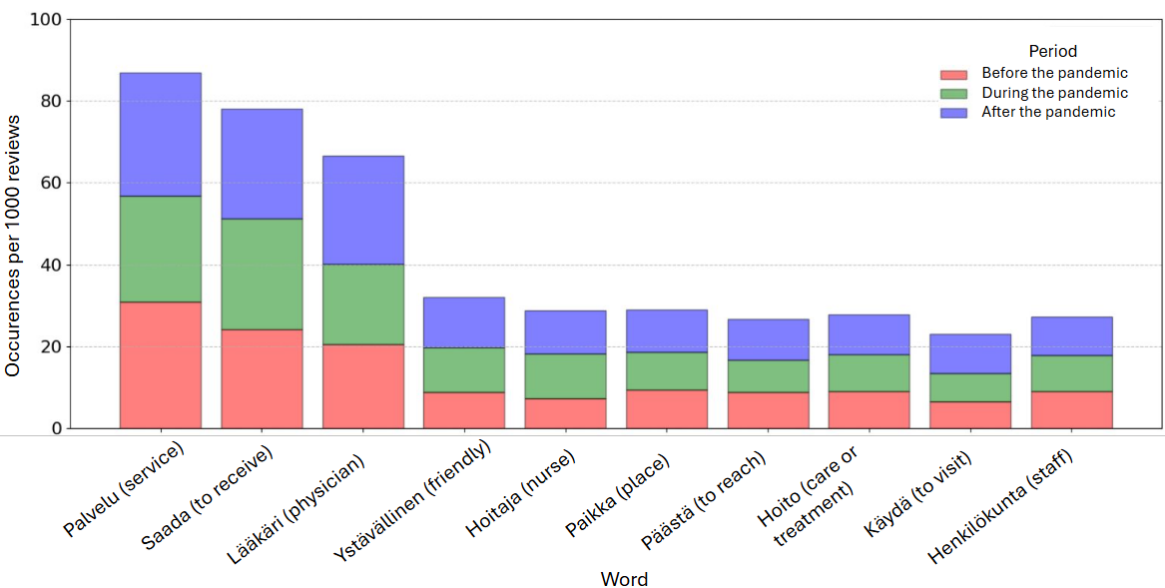
Relative frequency of the top words in Finnish-language reviews (per 1000 reviews) before, during, and after the pandemic.

Next, we present an analysis of overall word frequencies identified within the Spanish-language (Table 12) and Finnish-language (Table 13) reviews across the 3 defined periods. The tables report the relative frequencies of each word by normalizing the absolute counts by the number of reviews in each period. In addition, we report the occurrences for each of the 10 most frequent words per 1000 reviews for the Spanish (Figure 13) and Finnish (Figure 14) languages.

**Table 12:** The 10 most frequent words in Spanish-language reviews before, during, and after the pandemic.

Ranking	Before the pandemic	During the pandemic	After the pandemic
1	“Centro” (center; 27.11%)	“Teléfono” (telephone; 40.89%)	“Centro” (center; 43.65%)
2	“Médico” (physician; 19.78%)	“Centro” (center; 40.18%)	“Cita” (appointment; 41.14%)
3	“Salud” (health; 19.13%)	“Cita” (appointment; 34.05%)	“Médico” (physician; 35.16%)
4	“Atender” (to attend or assist; 17.96%)	“Coger” (to pick up or answer; 31.45%)	“Salud” (health; 31.44%)
5	“Atención” (attention or care; 17.73%)	“Llamar” (to call; 29.81%)	“Atender” (to attend or assist; 30.88%)
6	“Teléfono” (telephone; 17.21%)	“Salud” (health; 29.58%)	“Teléfono” (telephone; 25.86%)
7	“Cita” (appointment; 15.75%)	“Atender” (to attend or assist; 29.4%)	“Urgencia” (emergency; 25.38%)
8	“Llamar” (to call; 13.68%)	“Médico” (physician; 26.13%)	“Coger” (to pick up or answer; 23.56%)
9	“Personal” (staff; 13.05%)	“Vergüenza” (shame; 18.99%)	“Persona” (person; 21.05%)
10	“Urgencia” (emergency; 12.85%)	“Atención” (attention or care; 18.85%)	“Llamar” (to call; 20.71%)

**Table 13:** The 10 most frequent words in Finnish-language reviews before, during, and after the pandemic.

Ranking	Before the pandemic	During the pandemic	After the pandemic
1	“Palvelu” (service; 30.87%)	“Saada” (to get or receive; 27.21%)	“Palvelu” (service; 30.01%)
2	“Saada” (to get or receive; 24.14%)	“Palvelu” (service; 26.03%)	“Saada” (to get or receive; 26.74%)
3	“Lääkäri” (physician; 20.64%)	“Lääkäri” (physician; 19.46%)	“Lääkäri” (physician; 26.56%)
4	“Paikka” (place; 9.41%)	“Ystävällinen” (friendly; 10.96%)	“Ystävällinen” (friendly; 12.24%)
5	“Hoito” (care or treatment; 9.04%)	“Hoitaja” (nurse; 10.82%)	“Hoitaja” (nurse; 10.7%)
6	“Henkilökunta” (staff; 9%)	“Paikka” (place; 9.26%)	“Paikka” (place; 10.34%)
7	“Päästä” (to get to or reach; 8.91%)	“Hoito” (care or treatment; 8.97%)	“Päästä” (to get to or reach; 9.88%)
8	“Ystävällinen” (friendly; 8.83%)	“Toimia” (to work or function; 8.88%)	“Hoito” (care or treatment; 9.79%)
9	“Toimia” (to work or function; 7.51%)	“Soittaa” (to call; 8.88%)	“Käydä” (to visit or go; 9.7%)
10	“Hoitaja” (nurse; 7.35%)	“Henkilökunta” (staff; 8.79%)	“Henkilökunta” (staff; 9.43%)

We can observe in Table 12 that certain words such as “centro” (center), “médico” (physician), “salud” (health), “atender” (to attend or assist), “urgencia” (emergency), “persona” (person), “personal” (staff), and “atención” (attention or care) appeared consistently across all 3 periods, although their frequency fluctuated. A change was observed during the pandemic regarding the frequency of words related to remote communication with the health center.

As shown in Figure 13, the frequency of words related to phone communication—“teléfono” (telephone), “coger” (to pick up), and “llamar” (to call)—increased significantly during the pandemic before declining afterward. In contrast, the frequency of the word “cita” (appointment) not only saw a sharp rise during the pandemic but also continued to increase even further in the postpandemic period. The frequency of the word “vergüenza” (shame) also experienced a notable increase during the pandemic, and although it decreased slightly after the pandemic, it remained much higher than prepandemic levels.

For the Finnish language, we can observe in Table 13 that the most frequent words revealed persistent themes across the periods. Words related to health care delivery, such as “palvelu” (service), “saada” (to get or receive), and “lääkäri” (physician), consistently ranked among the top 3 terms, although their relative order varied.

Interestingly, we found a difference regarding specific socioemotional terms in both regions. In Andalusia, we observed a rise in the use of the word “vergüenza” (shame), entering the top 10 words (position 9) during the pandemic. Conversely, in Finland, the adjective “ystävällinen” (friendly) moved from the eighth most used term to the fourth most used term overall.

Examples of extremely positive and negative Spanish- and Finnish-language reviews translated into English and anonymized are shown next, the Finnish language examples in Table 14, along with the sentiment scores obtained through both the lexicon- and deep learning–based methods.

**Table 14:** Examples of extremely positive and negative reviews translated from Finnish. The words that appear in the list of the 10 most common words in Figure 14 are marked in italics.

Finnish-language review text (translated and anonymized)	Lexicon-based sentiment score	Deep learning–based sentiment score
“Today 15.09.2023 I went to the [pharmacy name] and bought my medication, and at the same time three prescriptions should be renewed (at about 14.00). At 18.00 I got a SMS that the prescriptions have been renewed! BIG THANKS TO THE [health center name] AND THE *DOCTOR*!”	24	1
“A calm and clean environment, where *staff* walking in the corridors greets the patients. A nice little café with the smell of buns.”	23	1
“Really bad *services*, the *nurses* who answered the phone treated me inappropriately and rudely. This is not the first time, fed up with [health station name] *services*. I have an urgent and emergency situation, but they did not give me an earlier time, I should wait a month and a half then I have an appointment with the *doctor*. Does not value patient rights. Did not understand that pain/illness is making my working life difficult and should be treated as soon as possible, they do not care.”	−14	–1
“According to the callback system, the *nurse* called, but answered as dully as can be. I almost wept, as I was feeling terrible anyways. Suspected corona. No points for the [health care center 1]. Things are handled 100 times better in [health care center 2]. I’ve never been offended there. The quality of *service* in [health care center 1] is second to none. In other words, a lottery. It is very annoying to come to their health care again. The older you get, the worse the *care* you get. Scary. Why hasn’t this style changed in [health care center 1] for over 20 years?? I’m just asking!”	−13	–1

In the following is an example of an extremely positive review translated from Spanish. The words that appear in the list of the 10 most common words in Table 12 are marked in italics. The lexicon-based sentiment score is 27, the deep learning–based sentiment score is 1, and the review text is: “*Doctor* [person name 1] is wonderful, polite, kind, caring, generous, and a great medical professional. Without a doubt, one of the very best in all [province name]. Her nurse, [person name 2], is also lovely and caring. I love this *health center* because you leave with the feeling that you’ve been healed. A ten out of ten score. They are all incredible workers. A pleasure dealing with them.”

In the following is an example of an extremely negative review translated from Spanish. The words that appear in the list of the 10 most common words in Table 12 are marked in italics. The lexicon-based sentiment score is –32, the deep learning–based sentiment score is –1, and the review text is “I’m giving it one star because I can’t give zero. The *doctors* are not to blame, but the *service* at the front desk is lamentable. They don’t know how to do their job, they can’t schedule an *appointment* correctly, they don’t even know how to use the computer program, and on top of that, the ladies treat you with appalling rudeness (or outrageous arrogance). They accept their lack of ability as something normal, and sadly, they have normalized it in their day-to-day work. The waste of time is continuous, and the time they make citizens waste who come to be seen is something that needs looking into. In our specific case, they have messed up an *appointment* four times for a well-child checkup. First, they got the doctor wrong, then they sent us to the nurse instead of the pediatrician, today the doctor didn’t show up and nobody notified us, and to top it all off, they give me a *telephone appointment* after I spent 20 minutes demanding a proper *appointment*...My daughter is 15 months old and hasn’t been seen by a pediatrician for 11 months. These ladies should have to demonstrate their competence for the position they hold, and if they don’t fulfill their duties or don’t know how to perform their job correctly, replacements who are capable of doing those tasks should be sought. It’s a public position that we all pay for, dependent on the Andalusian *Health Service*, and we citizens who come to be *attended* to should not have to waste our time like this. Shameful!”

## Discussion

### Principal Findings

This study showed how a systematic data analysis pipeline can be built for monitoring the perceived quality of primary health care using UGC. The analysis pipeline was tested using Google Maps reviews from primary health care locations in Finland and Andalusia, Spain. Similar approaches have not been previously implemented for Finland or Spain. Google Maps reviews have traditionally been mostly used in assessing perceived service quality in the hospitality industry, but exploiting them in the domain of health care is still in its infancy. There is some previous work in Asian countries such as Taiwan [17] and Malaysia [18] but not in Europe. However, mining user-generated social media data is a cost-effective way of monitoring perceived service quality when compared to surveys [23].

In addition to showing how the data analytics pipeline can be built, we found that the COVID-19 pandemic did remarkably affect the perceived quality of service of primary health care in Andalusia. As shown in Tables 10 and 11, the median values for the sentiment analysis of the textual reviews were neutral before the pandemic. However, both during and after the pandemic, the median of the lexicon-based sentiment analysis results was –2 (IQR 1), and the median of the results of the sentiment analysis conducted using the deep learning–based model was –1. Regarding numeric user ratings for Andalusia, we observed similar changes for worse during and after the pandemic. The mean user ratings before the pandemic were remarkably higher than during and after, as can be observed in Figure 9 and Table 8. The number of reviews with the most positive rating (5) decreased since the pandemic began. This is in line with the findings of Lopez-Picazo et al [52], who presented results on inpatients’ perceived quality based on the hospital regular monitoring plan and the net promoter score in a Spanish hospital. They studied the perceptions before and during the COVID-19 pandemic and reported similar results to those of our study regarding Andalusia—the perceived quality of health care was remarkably lower during and after the pandemic than before it. The lexical analysis revealed important patterns in how health care experiences were described before, during, and after the pandemic. We found an increasing salience of terms related to access and communication as the pandemic progressed. In Spanish-language reviews, nouns such as “teléfono” (telephone) and “cita” (appointment) dominated negative feedback, especially during and after the pandemic, possibly reflecting dissatisfaction with remote communication and appointment systems or availability. Similarly, in Finnish-language reviews, the term “soittaa” (call by phone) appeared on the list of the 10 most frequent words only during the pandemic. For both languages, positive nouns related to service and staff remained highly frequent across periods, suggesting appreciation and acknowledgment.

This paper presents results of a longitudinal study on perceived service quality in health care and the possible effect of a large-scale event (the COVID-19 pandemic) on it. The body of work on this topic is scant, especially when it comes to studies based on sentiment analysis of UGC. The work by Lopez-Picazo et al [52] presents results on the perceived service quality in a Spanish hospital before and during the pandemic, but it is based on surveys. Ours is the first study to address changes in sentiment in UGC regarding health care services before, during, and after the COVID-19 pandemic in Europe. The work by Aduragba et al [53] compares emotions in tweets geolocated in London during March 2019 and March 2020. They observed a rise in the negative emotions of sadness, anxiety, and annoyance when comparing 2020 to 2019. However, their work does not address health care service providers.

Another finding of our study was that the sentiments did not vary in Finland. The mean of both numeric user ratings and sentiment in reviews stayed the same before, during, and after the pandemic. There were very modest differences in the mean values and in minima and maxima. The results concerning the perceived quality of health care in Finland are new and interesting as there is no previous research in that domain. From the publicly available customer satisfaction data collected by Finnish primary care organizers, we know that 77% of the customers completely agree with the following statement: “I received the service/treatment when I needed it” [54]. Moreover, data from Finland show how access to care improved after the outbreak of COVID-19. In October 2019, a total of 40% of nonurgent physician’s appointments in primary health care happened within <7 days. In March 2024, the figure was already almost 60% [55]. In Andalusia, on the contrary, the mean waiting time to access primary health care tripled since December 2018. In 2018, the mean waiting time was 3.8 days, and in 2023, it was 10.4 days [56]. We know that, in Spain and many European countries, the perceived quality of health care decreased during and after the pandemic [52,57], and we know that, according to the study by Tuczyńska et al [57], the perceived quality has remained relatively stable in the United Kingdom. The work by Ainley et al [9] shows results for the United Kingdom along the same lines. However, their work focuses on the delivery of remote care, and the data were analyzed only until October 2020. Tuczyńska et al [57] studied the changes in perceived quality of health care services in 8 European countries. They report a decrease in all the studied countries except the United Kingdom. However, Finland and Andalusia were not addressed in their study.

### Limitations

This study has several limitations. First, we did not control for overall change in ratings and sentiment in Google Maps data for Finland or Andalusia. Thus, we do not know if, for example, the ratings and sentiment for all locations in Andalusia dropped since the beginning of the pandemic. However, previous studies using data from other locations do not show an overall drop in sentiment. A previous study by Aunimo and Martin-Domingo [58] assessed the overall sentiment in UGC on airport services before and during COVID-19 pandemic, and the results showed that the overall sentiment stayed almost the same with a slight increase and only COVID-19–related content showed a decrease in sentiment. The study by Ainley et al [9] analyzed user sentiment toward access to and quality of remote care and noted an increase in positive sentiment in the time after the COVID-19 outbreak in comparison to the time before. However, it would be beneficial if a future study analyzed either the overall sentiment of Google Maps data before, during, and after the COVID-19 pandemic in Andalusia or the sentiment regarding other services such as hotel and travel or public schools to investigate the extent of the drop in sentiment in the area.

Another limitation of our study is that we did not control for possible changes in sentiment due to other simultaneous transformations in the health care systems. For instance, in Finland, there was a major reorganization of the entire public health care system on January 1, 2023. This major change is observed in the last part of the data for the period during the COVID-19 pandemic and in the entire dataset for the period after the COVID-19 pandemic. However, the key primary health care providers (such as health centers or *terveyskeskus* and health stations or *terveysasema*) stayed the same, and some differences in their names were compensated by the semantic search mechanism on Google Maps. The collected dataset is consistent for the purpose of this analysis.

A third possible limitation is that, while user ratings and reviews are very popular for some locations such as restaurants and hotels, Google Maps may not be as popular a forum for patients to express their sentiment regarding health care service quality. This may hold true especially for patients in Finland, where the number of reviews in proportion to the population was lower than that for Andalusia. Finland has 5.5 million inhabitants and 12,252 user ratings, whereas Andalusia has 8.5 million inhabitants and 42,796 user ratings. One reason for a lower number of ratings and reviews could be that some important locations such as schools that also provide public primary health care services were left out of the Finnish dataset. Another reason for a lower number of Finnish ratings and reviews may be that the working population typically does not use the public primary health care services as their primary health care is organized through occupational health care services in private health care centers. Thus, demographic characteristics such as age, gender, socioeconomic status, and area of residence may be biased in Google Maps reviews. Moreover, existing studies show that individuals who leave reviews often have more extreme experiences—either highly positive or highly negative—leading to potential bias in the dataset [59]. In addition, older patients (who may be less internet active) are underrepresented [60]. However, user-generated data are not meant to replace official surveys conducted by health care organizations but, rather, are meant to complement the data. Moreover, the aforementioned skews [59,60] do not affect our main research question—sentiment change over time (before, during, and after the COVID-19 pandemic).

### Conclusions

This study demonstrated a considerable change in a negative direction in the sentiment of public discussions on primary health care in Andalusia starting from the beginning of the COVID-19 pandemic. However, no change was observed in the sentiment of the corresponding public discussions in Finland. This can be observed in both the numeric user ratings and the sentiment analysis of the review texts. The second finding of this study is that there is a positive correlation between numeric ratings and sentiment analysis of textual reviews, confirming the reliability of the sentiment analysis tools used. Third, we illustrated how Google Maps reviews may be used to monitor public opinion on primary health care. This study provides novel information on the effect of the COVID-19 pandemic on the perceived service quality in primary health care. It also provides insight into how Google Maps reviews may be used to monitor the sentiment of reviews related to hundreds of primary health care locations. Previous research using Google Maps reviews has typically focused on a single location with many reviews, such as a university hospital. Our study presents a methodology for identifying primary health care locations in a specified area and subsequently processing reviews related to them. Further investigations should be conducted to find out why the perceived service quality of primary health care measured using UGC in Andalusia shifted considerably toward negative sentiment since the start of the COVID-19 pandemic and why this was not the case for Finland. For example, analysis of policy response measures reveals that these were stricter in Spain throughout the pandemic (see the COVID-19 stringency index comparison in Figure 2). The impact of other factors such as culture and efficiency of health systems [61] should also be analyzed.

## Abbreviations

UGC: user-generated content
WHO: World Health Organization

## Data Availability

The datasets generated or analyzed during this study are not publicly available due to the restriction on creating content based on Google Maps content but are available from the corresponding author on reasonable request for noncommercial use.
